# Photosynthetic Characteristics and Chloroplast Ultrastructure Responses of Citrus Leaves to Copper Toxicity Induced by Bordeaux Mixture in Greenhouse

**DOI:** 10.3390/ijms23179835

**Published:** 2022-08-30

**Authors:** Fei Lu, Pingping Hu, Meilan Lin, Xin Ye, Lisong Chen, Zengrong Huang

**Affiliations:** College of Resources and Environment, Fujian Agriculture and Forestry University, Fuzhou 350002, China

**Keywords:** citrus, Bordeaux mixture, photosynthesis, Cu toxicity, ultrastructure

## Abstract

Bordeaux mixture (Bm) is a copper (Cu)-based pesticide that has been widely used for controlling citrus scab and citrus canker. However, frequent spraying of Bm is toxic to citrus. To our knowledge, few studies are available that discuss how the photosynthetic characteristics and chloroplast ultrastructure of citrus leaves are affected by Cu toxicity induced by excessive Bm. In the study, two-year-old seedlings of *Citrus grandis* (*C. grandis*) and *Citrus sinensis* (*C. sinensis*), which were precultured in pots, were foliar-sprayed with deionized water (as control) or Bm diluted 500-fold at intervals of 7 days for 6 times (4 times as recommended by the manufacturer) to investigate the leaf Cu absorption, photosynthesis, chloroplast ultrastructure and antioxidant enzymatic activities. Bm foliar-sprayed 6 times on citrus seedlings increased the leaf Cu content, decreased the photosynthetic pigments content and destroyed the chloroplast ultrastructure, which induced leaf chlorosis and photosynthetic inhibition. A lower Cu absorption, a higher light photon-electron transfer efficiency, a relative integrity of chloroplast ultrastructure and a promoted antioxidant protection contributed to a higher photosynthetic activity of *C. grandis* than *C. sinensis* under excessive spraying of Bm. The present study provides crucial references for screening and selecting citrus species with a higher tolerance to Cu toxicity induced by excessive Bm.

## 1. Introduction

Bordeaux mixture (Bm), containing copper (Cu) and lime, has been extensively used for disease control of fruit crops, such as apple leaf spot [1], mango apical necrosis [2] and olive fruit fly [3]. The biocidal action of Bm is based on the combination of Cu^2+^ with pathogenic proteins, which desaturated and inactivated the intracellular enzymes. Because of its broad spectrum, high efficacy and low cost in pathogen controlling, Bm was recognized as one of the most conventional fungicides in fruit crop production [4]. In citrus orchard management, Bm was frequently sprayed for the prevention and control of citrus scab and citrus canker for more than 100 years [5]. Consequently, the Bm solution dripped from the citrus leaves accumulated on the topsoil of citrus orchards year-by-year, leading to an excess amount of available Cu in the soil [6,7]. For instance, the investigation of mineral nutrition condition in the soils and leaves of pummelo groves in the Pinghe county of Fujian Province in south China have indicated that the ratio of available Cu that represents super-optimal levels in the leaves (>17 mg·kg^−1^) and the soils (>6 mg·kg^−^^1^) increased from 41.0% to 70.3% and 5.0% to 28.3% from 2001 [8] to 2011 [9], respectively. Similar findings were reported in the vineyards [10] and apple orchards [11].

Excessive Cu inhibits the photosynthesis of citrus leaves. To our knowledge, the existing reports regarding the effects of Cu stress on the photosynthetic characteristics of citrus species was mainly carried out hydroponically or by sandy culture. For example, Zhang et al. [12] discussed the effects of Cu levels on the leaf photosynthesis of navel orange seedlings under 5–40 μM Cu^2+^ stress lasting for 3 months hydroponically. Differentially, Li et al. [13] explored the effects of Cu^2+^ toxicity from 0.5–500 μM on citrus photosynthesis over a 6 month duration in sandy culture. The Cu translocation in citrus species of the studies mentioned above depends on root absorption. Although similar conclusions have been drawn from the studies mentioned above, the effects of foliar-applied Cu on the photosynthesis of citrus leaves are not neglectable. In agronomic practices, the Cu translocation into citrus leaves through foliar absorption from Cu-plus fungicides, such as Bm, was supposed to be more direct and prompter than Cu uptake from the rhizosphere [14]. Accordingly, the leaf Cu toxicity induced by excessive foliar-spray of Bm is often acute and the symptom of leaf Cu toxicity is much more apparent. The responses of citrus leaves to Cu toxicity induced by excessive Bm might also differ from the studies mentioned above. However, little information is available on the photosynthetic responses to Cu toxicity induced by excessive Bm in citrus species. Understanding the phytotoxicity of over-spraying Bm on citrus species is significant for a reference on the rational application of Bm. Moreover, revealing the responses of citrus species to excessive Bm is also crucial for screening citrus species with a better photosynthetic performance in citrus breeding.

The study aimed to investigate how the photosynthetic characteristics and chloroplast ultrastructure of citrus leaves are affected by copper toxicity induced by excessive Bm in seedlings of ‘Shatian pummelo’ [*Citrus grandis* (L.) Osbeck] and ‘Xuegan’ [*Citrus sinensis* (L.) Osbeck]. Two-year-old seedlings of *C. grandis* and *C. sinensis* were sprayed with deionized water (as control) or Bm diluted 500-fold at intervals of 7 days for 6 times. At the end of treatments, leaf Cu concentration, photosynthetic pigments content, gas exchange, the chlorophyll (*Chl*) a fluorescence transients, the ultrastructure of leaf chloroplast, the leaf electrolyte leakage rate (ELR) and activities of antioxidant enzymes [superoxide dismutase (SOD), peroxidase (POD) and catalase (CAT)] were investigated. The present study’s findings could shed light on the physiological responses of citrus leaves to Cu toxicity induced by excessive Bm and provide crucial references for screening and selecting citrus species with a higher tolerance under Cu toxicity.

## 2. Results

### 2.1. Effects of Excessive Spraying of Bm on the Leaf Morphology and Cu Content of Two Citrus Species

**Figure 1:**
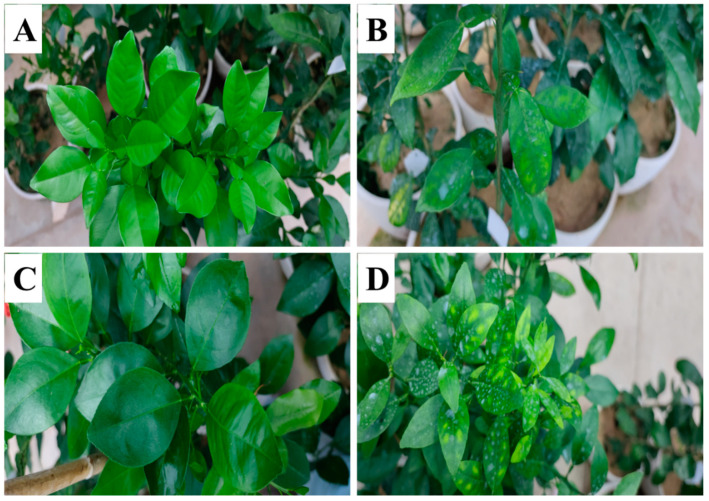
The effects of excessive spraying of Bordeaux mixture (Bm) on the leaves of two citrus species. (**A**) control *C. grandis*; (**B**) excessive Bm-treated *C. grandis*; (**C**) control *C. sinensis*; (**D**) excessive Bm-treated *C. sinensis*.

**Figure 2:**
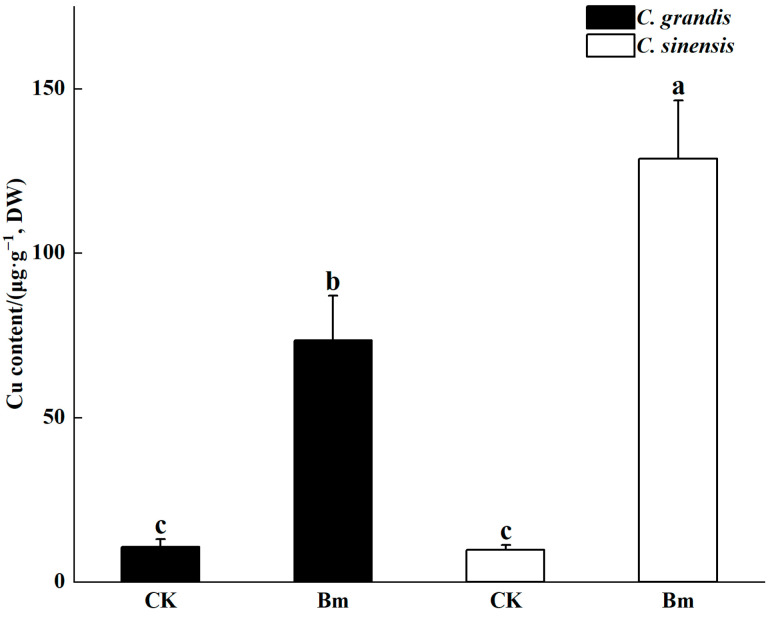
The effects of excessive spraying of Bordeaux mixture (Bm) on the leaf Cu content of two citrus species. Values represent means ± SE, *n* = 5. Significant differences (*p* ≤ 0.05) between treatments are indicated by different letters.

The control leaves of two citrus species were healthy, and the color of the leaf surface was bright green without any yellowing (Figure 1A,C). However, excessive spraying of Bm formed a whitish dry residue on the leaves and induced prominent necrotic spots between leaf veins of two citrus species (Figure 1B,D). The younger leaves suffered much more than the lower leaves of citrus seedlings that received excessive Bm. Moreover, the leaf chlorosis induced by excessive Bm was much more severe in *C. sinensis* than in *C. grandis*. Excessive spraying of Bm significantly increased the Cu accumulated in the citrus leaves compared to the control. As found in Figure 2, the Cu level in the leaves receiving excessive Bm was 5.9 and 12.2 times higher than in that of control in *C. grandis* and *C. sinensis*, respectively. Strikingly, the Cu content in *C. sinensis* leaves was significantly higher (*p* = 0.04) than that in *C. grandis* by excessive spraying of Bm.

### 2.2. Effects of Excessive Spraying of Bm on the Leaf Photosynthetic Pigments of Two Citrus Species

**Figure 3:**
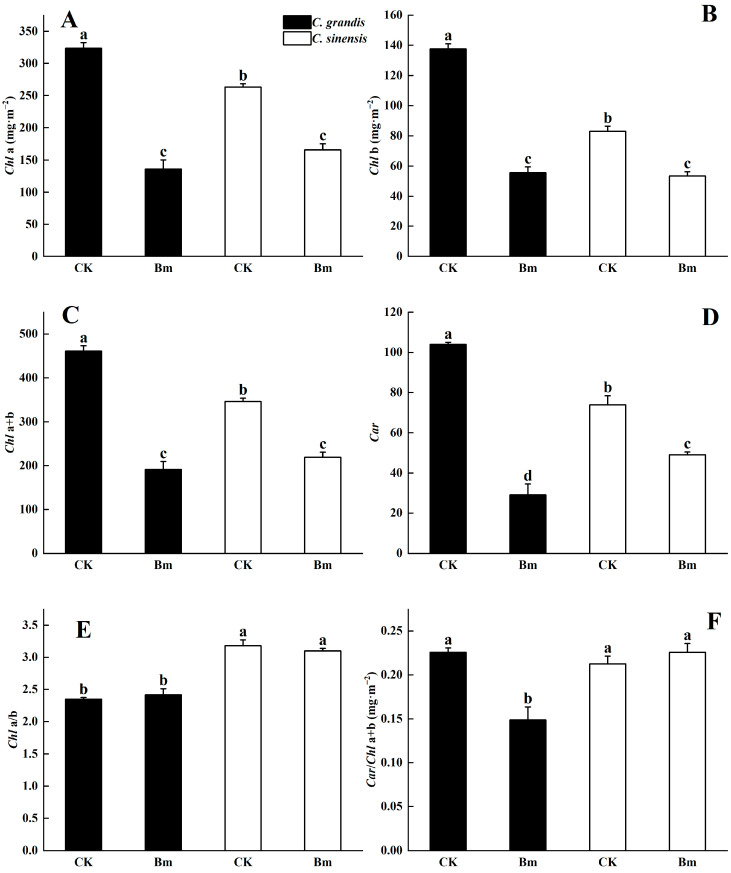
The effects of excessive spraying of Bordeaux mixture (Bm) on the photosynthetic pigments of two citrus species. (**A**) *Chl a*; (**B**) *Chl b*; (**C**) *Chl a* + *b*; (**D**) *Car*; (**E**) *Chl a*/*b*; (**F**) *Car*/*Chl a* + *b*. Values represent means ± SE, *n* = 5. Significant differences (*p* ≤ 0.05) between treatments were indicated by different letters.

Excessive spraying of Bm remarkably decreased the contents of *Chl a* (Figure 3A), *Chl b* (Figure 3B), *Chl a* + *b* (Figure 3C) and carotenoids (*Car*) (Figure 3D) in the leaves of two citrus species. The *Chl a*, *Chl b* and *Car* levels of the excessively sprayed Bm leaves decreased by 55.0%, 57.7% and 69.2% in *C. grandis*, and 37.1%, 35.7% and 35.5% in *C. sinensis* compared to the control leaves, respectively. Under the treatment of excessive Bm, *C. sinensis* had significantly higher *Car* (*p* = 0.01), *Chl a*/*Chl b* (*p* = 0.0002) and *Car*/*Chl a + b* (*p* = 0.007) than *C. grandis* (Figure 3E,F).

### 2.3. Effects of Excessive Spraying of Bm on the Leaf Gas Exchange of Two Citrus Species

**Figure 4:**
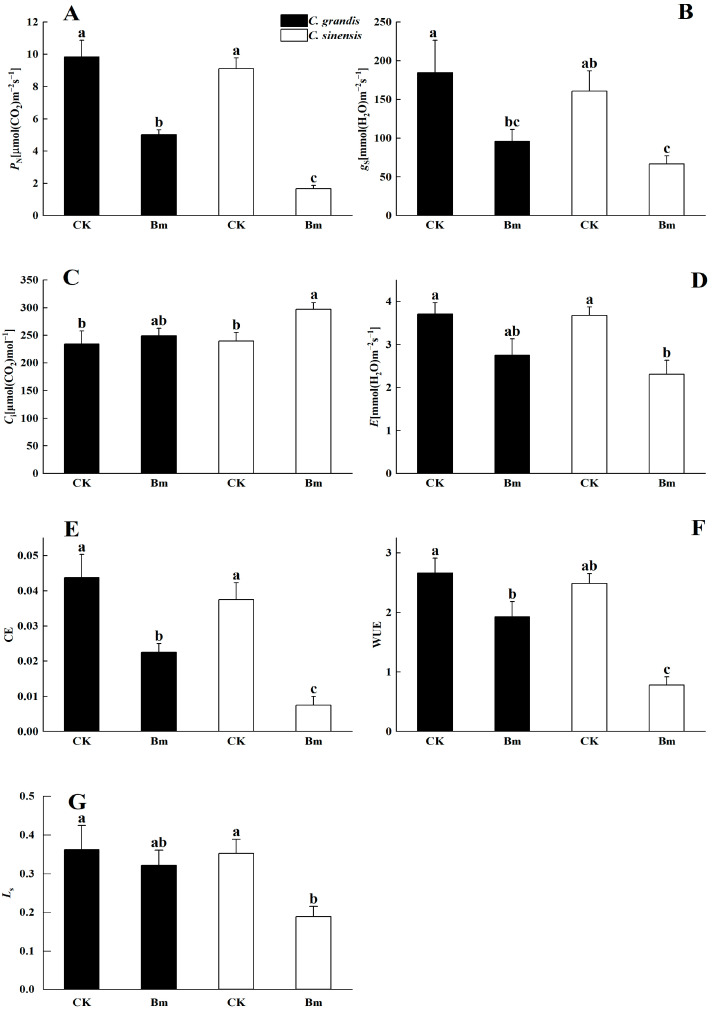
The effects of excessive spraying of Bordeaux mixture (Bm) on the leaf gas exchange of two citrus species. (**A**) *P*_N_, net photosynthetic rate; (**B**) *g*_s_, stomatal conductance; (**C**) *C*_i_, intercellular CO_2_ concentration; (**D**) *E*, transpiration rate; (**E**) CE, the carboxylation efficiency, CE = *P*_N_/*C*_i_; (**F**) WUE, water-use efficiency, WUE = *P*_N_/*E*; (**G**) *L*s, limitation of stomatal, *L*s = 1 − *C*_i_/C_a_. Values represent means ± SE, *n* = 10. Significant differences (*p* ≤ 0.05) between treatments are indicated by different letters.

The net photosynthetic rate (*P*_N_) was significantly decreased by 49.0% and 81.6% in *C. grandis* and *C. sinensis* leaves, respectively, after being excessively sprayed by Bm (Figure 4A). Significant downregulation of stomatal conductance (*Gs*), carboxylation efficiency of Rubisco (*CE*) and water-use efficiency (*WUE*) were also found in two citrus species under excessive Bm (Figure 4B–F). Differentially, the intercellular CO_2_ (*C*_i_) was only increased significantly (*p* = 0.02) in *C. sinensis* under excessive Bm compared to the control (Figure 4C,D). The significant decrease in transpiration rate (*E*, *p* = 0.01) and stomata limiting value (*Ls*, *p* = 0.01) was also only observed in *C. sinensis* but not in *C. grandis* under excessive Bm (Figure 4G).

### 2.4. Effects of Excessive Spraying of Bm on the Leaf Chl a Fluorescence (OJIP) Transients of Two Citrus Species

**Figure 5:**
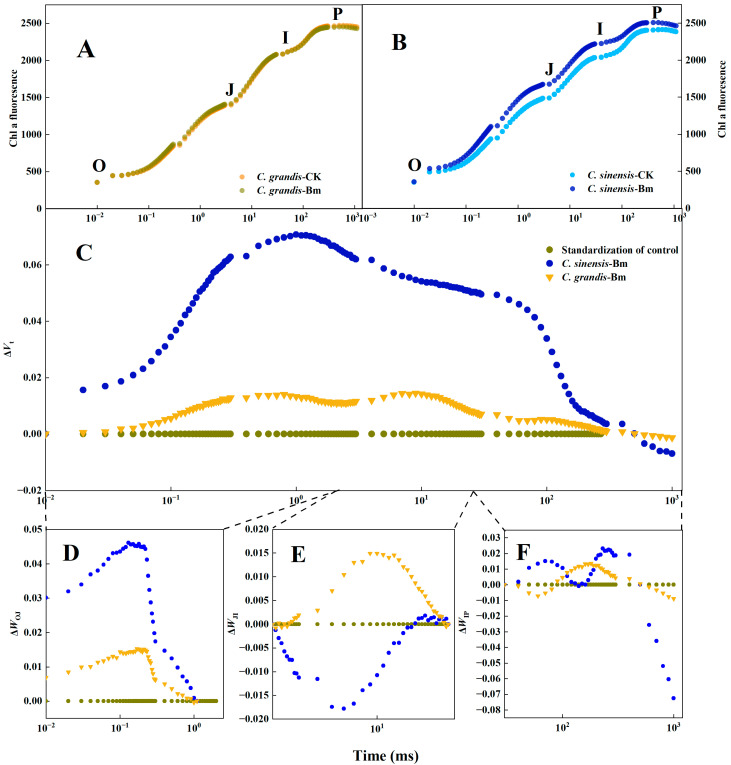
The effects of excessive spraying of Bordeaux mixture (Bm) on the leaf *Chl a* fluorescence transients of two citrus species. (**A**) *Chl a* of *C. grandis*; (**B**) *Chl a* of *C. sinensis*; (**C**) Δ*V*_t_; *V*_t_ =(F_t_ − F_0_)/(F_m_ − F_0_); Δ*V*_t_ = *V*_Bm_ − *V*_Control_; (**D**) Δ*W*_OJ_; *W*_OJ_ = (F_t_ − F_0_)/(F_J_ − F_0_); Δ*W*_OJ_ = *W*_OJ-Bm_ − *W*_OJ-Control_; (**E**) Δ*W*_OJ_; *W*_JI_ = (F_t_ − F_J_)/(F_I_ − F_J_); Δ*W*_JI_ = *W*_JI-Bm_ − *W*_JI-Control_; (**F**) Δ*W*_IP_; *W*_IP_ = (F_t_ − F_I_)/(F_m_ − F_I_); Δ*W*_IP_ = *W*_IP-Bm_ − *W*_IP-Control_; Values represent means ± SE, *n* = 10.

The fast *Chl a* fluorescence transients supported a more evident rising curve under excessive Bm compared to the control in *C. sinensis* leaves than that of *C. grandis* (Figure 5A,B). The rise is much more striking at the standardized curve (Δ*V*t) in Figure 5C. Furthermore, the curve was renormalized at the time course from 0.01–2 ms (O–J phase, Figure 5D), 2–30 ms (J–I phase, Figure 5D) and 30–1000 ms (I–J phase, Figure 5E). As shown in Figure 5D, both citrus species had a positive Δ*W*_OJ_ under excessive Bm compared to the control, which was higher in *C. sinensis*. However, the two citrus species had an overall adverse Δ*W*_JI_ (Figure 5E). Being treated with excessive Bm, most Δ*W*_JI_ in *C. sinensis* was positive, while most Δ*W*_JI_ in *C. grandis* was negative compared to the control. Much fluctuation in the Δ*W*_IP_ was found compared to Δ*W*_OJ_ and Δ*W*_JI_ (Figure 5F). Compared to *C. sinensis* treated with excessive Bm, the absolute value in Δ*W*_IP_ of *C. grandis* was lower than that of *C. sinensis,* except for the partial curve from 110 ms to 190 ms.

**Figure 6:**
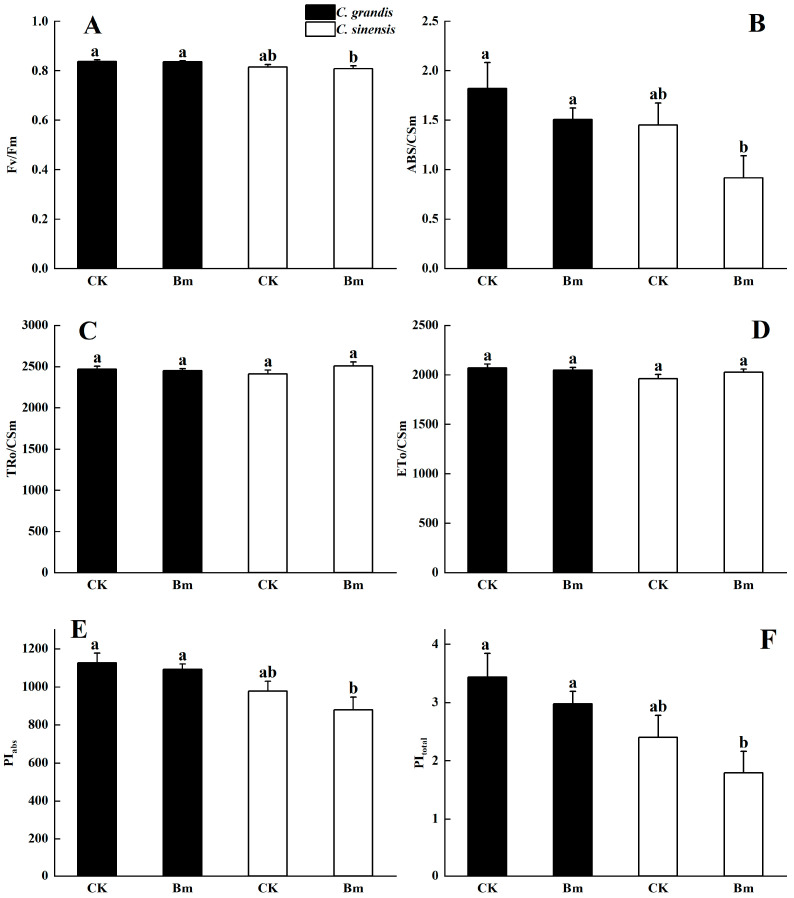
The effects of excessive spraying of Bordeaux mixture (Bm) on the leaf chlorophyll fluorescence parameters of two citrus species. (**A**) F_v_/F_m_; (**B**) ABS/CS_m_, light energy absorbed per active reaction center of PS II; (**C**) TR_o_/CS_m_, light energy trapped per active reaction center of PS II; (**D**) ET_o_/CS_m_, light energy transported per active reaction center of PS II. (**E**) PI_abs_, performance index on the absorption basis; (**F**) PI_total_, photochemical performance index of PS II reaction center; Values represent means ± SE, *n* = 10. Significant differences (*p* ≤ 0.05) between treatments are indicated by different letters.

The results in Figure 6 indicate an insignificant downregulation of *Chl a* fluorescence parameters affected by excessive Bm compared to the control in leaves of *C. grandis* and *C. sinensis*. However, the comparison between the two citrus species under excessive Bm indicated that *C. grandis* had a remarkable higher maximum photochemical efficiency of PS II (F_v_/F_m_, Figure 6A), the performance index of absorption (PI_abs_, Figure 6E), the photochemical performance index of PS II reaction center (PI_total_, Figure 6F) and light energy absorbed per active reaction center of PS II (ABS/CS_m_) than *C. sinensis*.

### 2.5. Effects of Excessive Spraying of Bm on the Ultrastructure of Leaf Chloroplast of Two Citrus Species

**Figure 7:**
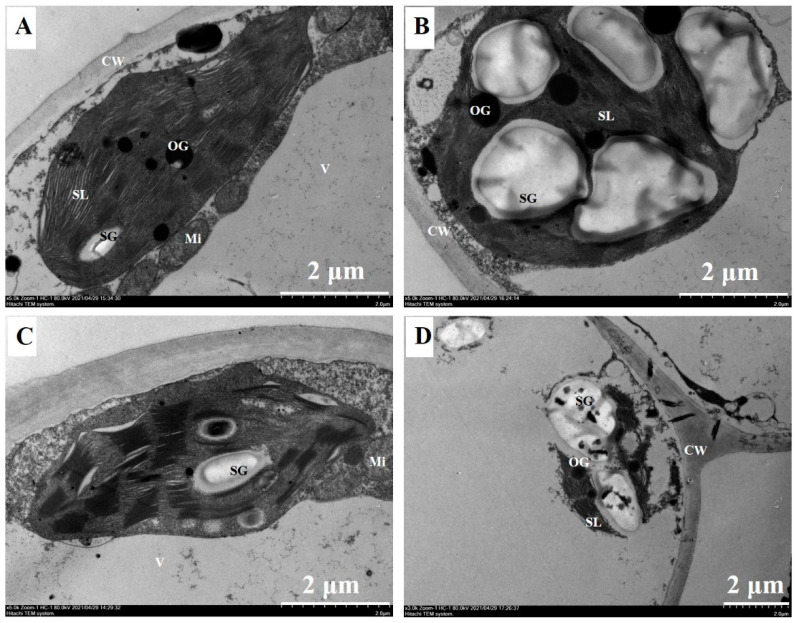
The effects of excessive spraying of Bordeaux mixture (Bm) on the ultrastructure of leaf chloroplast of two citrus species. (**A**) *C. grandis*-CK; (**B**) C. *grandis*-Bm; (**C**) *C. sinensis*-CK; (**D**) *C. sinensis*-Bm; CW: cell wall; Mi: mitochondrion; OG: osmiophilic globule; SG: starch grains; SL: stroma lamella; V: vacuole.

As found in Figure 7A,C, the structural integrity of the cell membrane was evident, and the fusiform chloroplasts of both citrus leaves were closed to the vacuole and adhered to the inner side of the cell wall without Bm spraying. The thylakoids inside the chloroplast were stacked in an orderly manner, and the lamellar structures were also apparent. Several osmiophilic globules and starch grains were distributed randomly inside the chloroplast. In contrast, excessive Bm significantly destroyed the bilayer structure of the leaf chloroplast membrane, loosened the lamellar structure of the thylakoid, and increased the number and volume of the starch grains in two citrus species (Figure 7B,D). Compared to *C. sinensis*, *C. grandis* maintained relative integrity of the chloroplast structure.

### 2.6. Effects of Excessive Spraying of Bm on the Leaf ELR and Activities of SOD, POD and CAT of Two Citrus Species

**Figure 8:**
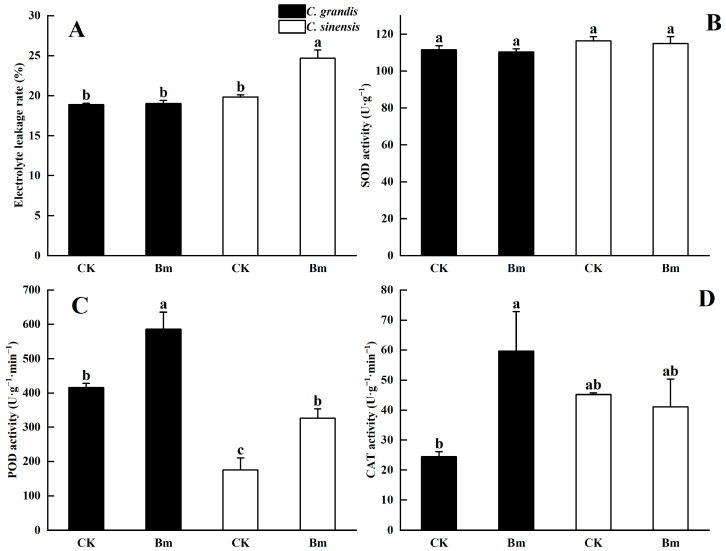
The effects of excessive Bordeaux mixture (Bm) on the leaf (**A**) electrolyte leakage rate and activities of (**B**) superoxide dismutase (SOD), (**C**) peroxidase (POD) and (**D**) catalase (CAT) of two citrus species. Values represent means ± SE, *n* = 5 for (**A**) and 4 for (**B**–**D**). Significant differences (*p* ≤ 0.05) between treatments are indicated by different letters.

Excessive Bm significantly increased the ELR in leaves of *C. sinensis* compared to the control. However, no significant increment was found in excessive Bm-treated *C. grandis* leaves compared to the control (Figure 8A). Under Bm treatment, *C. sinensis* had a remarkably higher ELR than *C. grandis*. Excessive Bm did not significantly affect the SOD activity in the two citrus species compared to the control (Figure 8B). A significantly higher POD activity than the control was observed in excessive Bm-treated leaves of the two citrus species. Under excessive Bm spraying, *C. grandis* had a significantly higher POD activity than *C. sinensis* (Figure 8C). Excess Bm significantly activated the CAT activity in *C. grandis* leaves. However, compared to the control, there was no significant increase in CAT activity in *C. sinensis* (Figure 8D).

## 3. Discussion

The photosynthetic performance of 300 μM Cu-treated *C. sinensis* and *C. grandis* seedlings under sandy culture has been explored in our previous report [15]. We revealed that *C. grandis* had a higher Cu accumulation in the lateral roots than *C. sinensis* when Cu was taken up from the roots. In the present study, excessive Cu was supplied by foliar application, which is traditional in agricultural practices, and resulted in a direct absorption of Cu and a much more apparent Cu toxic symptom of citrus leaves than Cu translocated from the roots to the leaves. Interestingly, the present results indicated that *C. grandis* leaves had less Cu accumulation than *C. sinensis*, which further supported a lower Cu translocation to the leaves of *C. grandis* than *C. sinensis* foliar supplied with excessive Cu. Despite the similar photosynthetic performance of the two citrus species under foliar-applied or sandy-watered Cu, the study further identified the ultrastructural alteration of chloroplast, which was highly correlated to the disordered photosynthetic processes under excessive Cu toxicity.

According to the manufacturer’s introduction, the Cu content of Bm wettable powder is approximately 23.2 ± 1.4%. Citrus leaves sprayed with Bm diluted 500–700-fold 4 times at intervals of 7 days are recommended for citrus canker prevention post-bloom when new shoots start sprouting (1–2 mm). During this study, we observed no visible phytotoxic symptom on the leaf surface of citrus seedlings after being sprayed with 500-fold diluted Bm (approximately 0.046% Cu^2+^) 4 times. However, leaf chlorosis appeared after citrus seedlings were foliar-sprayed with Bm 6 times. Accordingly, foliar-spraying of Bm 6 times at a 500-fold diluted concentration represented an excessive dose for *C. grandis* and *C. sinensis* seedlings in the study.

The symptom of Cu toxicity in citrus leaves induced by excessive spraying of Bm differed from that in sandy culture or hydroponics. Zhang et al. [12] reported that Cu toxicity of navel orange leaves in 20 μΜ Cu^2+^ hydroponically for 7 days induced chlorosis on the leaf tips. In our previous study, leaves of C. *grandis* and C. *sinensis* accumulated 40–50 μg·g^−1^ Cu^2+^ after being sandy cultured with 500 μM Cu (approximately 0.0032% Cu^2+^) for 6 months, which resulted in even leaf chlorosis in *C. grandis* and leaf wilting in *C. sinensis* [13]. In the present study, the Cu accumulated in the excessive Bm-treated leaves of C. *grandis* and C. *sinensis* were 73.5 μg·g^−1^ and 128.8 μg·g^−1^, respectively. The chlorosis between citrus leaf veins featured the phytotoxic symptom induced by excessive Bm. A similar symptom was reported in CuSO_4_-sprayed peach leaves [16]. The Cu content in the Cu-treated *C. sinensis* and *C. grandis* leaves of the studies mentioned above far exceeds the toxic threshold value (17.0 or 20.0 μg·g^−1^) [9,17]. Those different symptoms of Cu toxicity might be associated with the Cu tolerance of citrus species. Interestingly, *C. grandis* had a significantly lower Cu than *C. sinensis* in the leaves by foliar application of Bm. Furthermore, our previous finding [15] indicated that *C. grandis* had a significantly higher Cu in the lateral roots under sandy irrigation with Cu-plus solution. Those results together supported a higher Cu immobilization by the lateral roots and a lower Cu translocation by the leaves contributing to Cu tolerance of *C. grandis*.

Cu is indispensable for photosynthetic proteins, such as plastocyanin [18]. However, excessive Bm decreased *Chl a*, *Chl b* and *Car* content in citrus leaves (Figure 3), which was also identified in Bm-treated seedlings of *Vigna sinensis* and *Oryza sativa* [19]. The dramatic decrease in photosynthetic pigments was attributed to excessive Cu-induced oxidative stress [20]. It was found that in higher plants, *Chl a* and *Chl b* could be transformed reversibly from each through the *Chl* cycle [21]. Wang et al. [22] proposed a decreased *Chl a*/*Chl b* as an indicator of adaptation to decreased irradiance. Datta et al. [23] reported that a Cu nanoparticles-based antifungal pesticide decreased the *Chl a* content and increased *Chl b* in rice seedlings, which was supposed to be a protection for the light-harvesting complex under Cu stress. Moreover, Moutinho-Pereira et al. [24] reported that Bm-treated grapevine leaves had higher reflectance and transmitted less photon flux density than control leaves. In the study, excessive Bm-induced toxicity did not significantly downregulate the ratio of *Chl a*/*Chl b* compared to the control. In addition to *Chl a*/*Chl b*, the increased ratio of *Car*/*Chl a* + *b* was reported to be involved in photoprotection through thermal dissipation under stress [25]. Excessive Bm-treated *C. sinensis* had remarkably higher ratios of *Chl a*/*Chl b* and *Car*/*Chl a* + *b* than *C. grandis*, which implied a stronger photoprotection attributed to severe phytotoxicity from excessive Bm.

The leaf *P*_N_ was depressed significantly in the two citrus species that received excessive Bm compared to the control (Figure 4). Similarly, the CE and WUE were also inhibited dramatically, verifying a striking downregulated CO_2_ assimilation of the two citrus leaves by excessive Bm. The significant inhibition of photosynthesis was in agreement with the results in apple trees frequently treated with Bm during the growing season [26]. The stomatal limitation theory [27,28] in plant leaf gas exchange proposed that when the *P*_N_ inhibition was mainly attributed to non-stomatal limitation, such as leaf mesophyll cell injury under stress, the *C*_i_ increased (because of inhibited CO_2_ assimilation) while *g*_s_ and *L*s decreased. Contrastingly, when the stomatal limitation is the main reason for the *P*_N_ inhibition, such as the stomatal closure inducing less CO_2_ influx, *g*_s_ and *C*_i_ will decrease. It was found that excessive Bm-treated *C. sinensis* leaves had a significant decrease in *P*_N_ and *L*s accompanied by a significant increase in *C*_i_. However, in excessive Bm-treated leaves of *C. grandis*, the decrease in *P*_N_ and *g*_s_ did not induce a significant decrease in *C*_i_. Accordingly, the decrement in *P*_N_ in the excessive Bm-treated *C. sinensis* of the present study was supposed to be mainly induced by nonstomatal factors, such as photodamage and oxidation. No significant increase in *C*_i_ indicated that the gas exchange of *C. grandis* leaves might be less affected by Bm stress than *C. sinensis*. A similar finding was reported in Cu-treated *Limoniastrum monopetalum* [29].

The fast *Chl a* fluorescence transients (OJIP curve) analysis provides a non-invasive approach for diagnosing the alteration of the photosynthetic apparatus’s structure and function regarding the PS II reaction center, antenna pigment, electron donor and acceptor sides [30,31]. Heavy metal stress such as excessive cadmium [32], manganese [33] and zinc [34] remarkably affected the OJIP shape. Typically, a much-shifted OJIP curve suggested significantly more severe toxicity from excessive Bm. For instance, Mathur et al. [35] reported that an increasing level of chromium resulted in a much fluctuated OJIP curve compared to the control in wheat leaf, which was associated with the degree of leaf toxicity. Similar findings have been reported in Al-treated citrus leaves [36]. In the study, a more evident rising OJIP curve compared to the control was exhibited in the excessive Bm-treated leaves of *C. sinensis*, indicating a very pronounced toxicity from Bm (Figure 5A–C). The fluorescence at characteristic phases during photoelectron transferring could be further revealed in three individual phases at O–J (0–2 ms, Δ*W*_OJ_), J–I (0–30 ms, Δ*W*_JI_) and I–P (30–1000 ms, Δ*W*_IP_) after the renormalization of the OJIP curve. The appearance of a positive peak around 300 μs (K-band) in Δ*W*_OJ_ of Figure 5D indicates the imbalance of electron transport from the PS II donor side (oxygen-evolving complex, OEC) to the oxidized PS II reaction center of chlorophyll (P680^+^) and reduced PS II acceptors (Q_A_^−^), which is mainly associated with the inactivation OEC [37]. The negative J–I phase in Figure 5E characterized the decreased electron transferring rate from Q_A_^−^ to the plastoquinone pool [38]. The I–P phase implied the process of electron flowing from reduced plastoquinone (PQH_2_) to the electron acceptors side of the PS I end [39]. Consequently, Figure 5 implies that excessive Bm impaired the OEC of the two citrus species and decreased the electron transferring rate from donor to acceptor. Compared to *C. sinensis*, *C. grandis* maintained a relatively higher electron transferring rate from Q_A_^−^ to the plastoquinone pool under excessive Bm.

The *Chl a* fluorescence parameters were obtained from the OJIP curve, which provided a better understanding of the photochemical processes during electron transferring [40]. In the study, excessive Bm did not affect the maximal photochemical efficiency (F_v_/F_m_, Figure 6A) nor the light energy efficiency of absorbing (ABS/CS_m_, Figure 6B), trapping (TRo/CS_m_, Figure 6C) and transferring (ET_o_/CS_m_, Figure 6D) per reaction center of PS II. The performance index in the photochemical reaction of PS II (PI_abs_, Figure 6E) and in electron transferring from PS II to PS I (PI_total_, Figure 6F) in each citrus species compared to the control were not significantly decreased. Compared to the insignificant decrease in the fluorescence transients after dark adaptation, the above-mentioned significant inhibited leaf gas exchange might result from a potential light-stress in Bm-treated plants. However, under excessive spraying of Bm, *C. grandis* had a significantly higher F_v_/F_m_, ABS/CS_m_, PI_abs_ and PI_total_, implying its higher electron transferring efficiency than *C. sineneis*.

The chloroplast is the site for light energy harvest and transformation, which is sensitive to ion toxicity [41]. The present results, which show that excessive Bm impaired the integrity of the cellular structure and perturbed the lamellar structure of the thylakoid inside the chloroplast, were observed in the leaves of Cu-treated citrus [42] and rapeseed [43]. Compared to the control, the starch granules accumulated inside the chloroplast of excessive Bm-treated citrus leaves were attributed to an imbalanced metabolism of carbohydrates, which was also reported in citrus leaves that received phosphite, a novel fungicide for *Phytophthora* [44]. Moreover, the more apparent chloroplast disarrangement and a decreased concentration of Chl in excessive Bm-treated *C. grandis* indicated that the leaf senescence precedes *C. sinensis*.

The increased leaf ELR reflected a disrupted plasma membrane permeability [45]. It was reported that heavy metal toxicity such as excessive Pb, Zn and Cd significantly increased the ELR in needles of *Pinus halepensis* [46]. Moreover, it was also identified that Cu-sensitive jute varieties had significantly higher ELR than the Cu-resistant ones [47]. Excessive Bm induced a remarkably lower leaf ELR in *C. grandis* than *C. sinensis* (Figure 8A) coupled with the relative integrity of leaf ultrastructural alteration (Figure 7) as evidenced by a structural basis of *C. grandis* tolerating excessive Bm-induced toxicity.

Excessive Cu absorption induced oxidative damage of citrus leaves as a result of the over-accumulation of reactive oxygen species (ROS) [48]. Antioxidant enzymes such as SOD, POD and CAT played pivotal roles in ROS scavenging, alleviating oxidative stress [49]. It was evidenced that Cu toxicity significantly upregulated the enzymatic activities of SOD, POD and CAT in *Brassica napus* leaves [50] and flax shoots [51]. In the present study, excessive Bm-treated *C. grandis* leaves had a remarkably higher POD and CAT enzymatic activity (Figure 8C,D) than the control. However, *C. sinensis* only significantly increased POD activity under excessive Bm compared to the control. Those results manifested a promoted antioxidant protection of *C. grandis* contributing to Cu tolerance induced by excessive spraying of Bm.

## 4. Materials and Methods

### 4.1. Seedlings Culture and Treatments

In December 2018, fruits of *C. grandis* were harvested from Meizhou Academy of Agriculture and Forestry Sciences of Guangdong province. At the same time, fruits of *C. sinensis* were picked in a suburban citrus orchard in Fuzhou, Fujian province. All fruits of the two citrus species were stored under 4 °C until use. The seeds were germinated and cultured in a greenhouse of Fujian Agriculture and Forestry University, Jinshan Campus (26°5′ N, 119°14′ E), according to Lai et al. [52] in March 2019. After germination, uniformed-sized citrus seedlings were transplanted to ceramic pots pre-filled with clean river sand. The plants of each pot were watered every other day with 500 mL modified Hoagland nutrient (pH 6.8–7.0), containing 1 mmol·L^−1^ KNO_3_, 1 mmol·L^−1^ Ca(NO_3_)_2_, 0.1 mmol·L^−1^ KH_2_PO_4_, 0.5 mmol·L^−1^ MgSO_4_, 10 μmol·L^−1^ H_3_BO_3_, 2 μmol·L^−1^ MnCl_2_, 2 μmol·L^−1^ ZnSO_4_, 0.5 μmol·L^−1^ CuSO_4_, 0.065 μmol·L^−1^ (NH_4_)_6_Mo_7_O_24_ and 20 μmol·L^−1^ Fe-EDTA. In May 2021, two-year-old citrus seedlings were used for treatments after new twigs sprouted. The citrus seedlings were foliar-sprayed with 70 mL 500-fold diluted Bm (as Bm) or distilled water (as control) 6 times at intervals of 7 days. Each pot was covered with a plastic plate to avoid the drops of Bm falling into the pot during foliar application. There were 10 pots (2 seedlings of each pot) for each treatment of each citrus species. Then, 2 days after the end of treatments, 10 mature citrus leaves of each treatment were cleaned with distilled water to measure leaf gas exchange and *Chl a* fluorescence transients. Mature leaves were collected at a quarter height from the seedling top after being soaked in 0.5 mol·L^−1^EDTA-Na_2_ for 10 min to remove Bm residue and washed with distilled water 3 times. Some clean citrus leaves were dried with a paper towel and punched between leaf veins to obtain leaf discs for leaf photosynthetic pigments extraction and quantification. The other part of the clean leaves without leaf veins were cut into slices (about 3 mm × 5 mm) and fixed with 3% glutaraldehyde-1.5%paraformaldehyde solution (fixing solution) in 0.1 mol^−1^ phosphate buffer solution (PBS, pH = 7.2). The remaining leaves were sampled and dried under 70 °C for Cu quantification.

### 4.2. Cu Quantification of Citrus Leaves

Dried citrus leaves were ground and digested preliminarily in HNO_3_/HClO_4_ (5:1, *v*/*v*) overnight, followed by thorough digestion at 250 °C. The Cu content of the citrus leaves was assayed by PinAAcle 900F atomic absorption spectrometry (PerkinElmer Singapore Pte Let., Singapore) according to Li et al. [13]. There were 5 replicates of each treatment.

### 4.3. Extraction and Quantification of Photosynthetic Pigments of Citrus Leaves

Five leaf discs (0.2826 cm^2^) of each treatment were extracted with 6 mL 80% acetone in the dark overnight. The absorbance of the extracting solution was recorded at 470, 645 and 663 nm by a Libra S22 UV/Vis spectrophotometer (Biochrom *Ltd*., Cambridge, UK) according to Zhang et al. [36]. The content of *Chl a*, *Chl b* and *Car* were calculated as follows, according to Lichtenthaler [53]:

*Chl a* (mg·L^−1^) = 12.72 × A_663_ − 2.59 × A_645_;

*Chl b* (mg·L^−1^) = 22.88 × A_645_ − 4.76 × A_663_; (1)

*Chl a* + *b* (mg·L^−1^) = 20.29 × A_645_ + 8.05 × A_663_; (2)

*Car* (mg·L^−1^) = (1000 × A_470_ − 1.82 × *Chl a* − 85.02 × *Chl b*)/198 (3)

The unit of leaf pigments expressed by mg·L^−1^ was further transformed by mg·m^−2^, based on the area of leaf discs as follows:

Photosynthetic pigment concentration per square meter (mg·m^−2^) = Photosynthetic pigment concentration per liter (mg·L^−1^) × 10^−3^ × 6/(0.2826 × 5 × 10^4^). There were 5 replicates of each treatment.

### 4.4. Gas Exchange Measurement of Citrus Leaves

The net photosynthetic rate (*P*_N_), intercellular CO_2_ concentration (*C*_i_), transpiration rate (*E*), ambient CO_2_ concentration (*C*_a_) and stomatal conductance (*g_s_*) were recorded by the CIRAS-2 portable photosynthesis system (PP System, Herts, UK) according to Zhang et al. [54]. The measurement was conducted on a sunny morning from 10:00 to 11:00 under atmospheric pressure of 1010 ± 5 Pa, CO_2_ concentration of 380 µmol· mol^−1^, relative humidity of 60.3 ± 0.6%, leaf temperature of 30.6 ± 0.5 °C and light intensity of 1000 μmol·m^−2^·s^−^^1^. The carboxylation efficiency (CE = *P*_N_/*C*_i_), water-use efficiency (WUE = *P*_N_/E) and limitation of stomatal (*L*s = 1 − *C*_i_/*C*_a_) were calculated based on the recorded data. There were 10 replicates of each treatment.

### 4.5. Chl a Fluorescence (OJIP) Transients Analyses of Citrus Leaves

The leaf OJIP transient analyses were performed by a *Handy PEA* (*Hansatech Instruments Ltd.*, Norfolk, UK) after dark adaptation for 3 h at room temperature (approximately 28 ℃) according to Jiang et al. [55]. The recordings (F_t_) were normalized into relative variable fluorescence transients [*V*_t_ = (F *−* F_0_)/(F_m_ *−* F_t_)]. In order to make a comparison between treatments, *V*_t_ of Bm and CK were further normalized as Δ*V*_t_ = *V*_Bm_ − *V*_CK_, according to the method proposed by Tsimilli-Michael et al. [29]. Briefly, three individual characteristic phases at O–J [0–2 ms, *W_OJ_* = (F_t_ − F_0_)/(F_J_ − F_0_)], J–I [0–30 ms, *W_JI_* = (F_t_ − F_J_)/(F_I_ − F_J_)] and I–P [30–1000 ms, *W_IP_* = (F_t_ − F_I_)/(F_m_ − F_I_)] were renormalized by subtracting W_CK_ from W_Bm,_ respectively. The F_0_, F_m_, F_J_ and F_I_ of the above formulas were the minimal and the maximal fluorescence within 1000 ms, fluorescence at 2 ms and 30 ms, respectively. There were 10 replicates of each treatment.

### 4.6. Transmission Electron Microscopy (TEM) Analyses of Citrus Leaves

Sampling preparation and observation were performed according to Chen et al. [56] and Huang et al. [57] with minor modification. Briefly, citrus leaf samples were quickly vacuumed in the fixing solution in a syringe, followed by pre-fixing under 4 °C for 3 h. The pre-fixed samples were cut into several leaf blocks (about 1 mm × 1 mm) and rinsed by PBS 3 times at intervals of 15 min. The leaf blocks were then post-fixed with 1% OsO4-1.5% potassium hexacyanoferrate under 4 °C for 2 h, followed by rinsing with distilled water 3 times at intervals of 15 min. The fixed leaf samples were dehydrated in increasing ethanol concentrations of 30%, 50%, 70%, 80%, 90%, 95% and 100% (3 times) for 15 min. After dehydration, samples were rinsed with acetone twice at an interval of 10 min, replaced by a mixture of resin and acetone (*v*/*v* = 1:1) and infiltrated by a mixture of resin and acetone (*v*/*v* = 3:1) for 2 h on a shaker, respectively. The leaf blocks were dried with filter paper to remove acetone residue and stored in a basin overnight. Finally, each individual leaf block was embedded in Epoxy resin 618 and sliced into sections approximately 80 nm in thickness by an ultra-microtome (LEICA EM UC6, Wetzlar, Germany). The leaf sections were observed with a TEM (HITACHI HT7700, Tokyo, Japan) equipped with a digital camera after being stained with 2% uranyl acetate. There were 3 replicates of each treatment.

### 4.7. The ELR and Activities of SOD, POD and CAT of Citrus Leaves

The ELRs of citrus leaves were measured according to Long et al. [58]. Briefly, 20 leaf discs (0.2826 cm^2^) were transferred to a tube containing 50 mL distilled water and kept under dark at room temperature. The solution’s first electrical conductance (EC_1_) was recorded after 24 h. The second reading was recorded 15 min after incubation at boiling water (EC_2_). The ELR was calculated as: ELR (%) = EC_1_/EC_2_ × 100, where EC_1_ and EC_2_ represented an electrolyte leakage from the living and dead cells, respectively. There were 5 replicates of each treatment.

Crude extract of SOD, POD and CAT was prepared by homogenizing 0.03 g citrus leaves in 1.5 mL 50 mM PBS (pH = 7.8). The homogenate was centrifuged at 13,000× *g* for 10 min to collect the supernatant. The SOD activity was measured according to Giannopolitis and Ries [59]. The POD and CAT were monitored according to Onsa et al. [60] and Chen and Cheng [61], respectively. There were 4 replicates of each treatment.

### 4.8. Statistical Analysis

The data in this study were analyzed using SPSS (Version 22.0, IBM, Armonk, NY, USA). Values were means ± SE of 5 to 10 replicates. Four means (two treatments × two citrus species) were analyzed by two ANOVAs followed by Duncan’s multiple range test at *p* < 0.05.

## 5. Conclusions

Cu toxicity induced by foliar-spraying of Bm 6 times at a 500-fold diluted concentration in a greenhouse decreased the content of photosynthetic pigment and destroyed the chloroplast ultrastructure of two citrus species. The decrement in photosynthetic rate in *C. sinensis* under excessive spraying of Bm was mainly attributed to non-stomatal limitation. A lower Cu absorption, a higher light energy transfer efficiency, a relative integrity of chloroplast ultrastructure and a promoted antioxidant protection contributed to a higher photosynthetic activity of *C. grandis* than *C. sinensis* under excessive spraying of Bm. Compared to *C. sinensis*, *C. grandis* would be a potential species tolerant of excessive Bm.

## Data Availability

Data are archived in L.-S. Chen’s lab and are available upon request.
